# Effects of Opioids in Cancer Pain: An Interplay Among Genetic Factors, Immune Response, and Clinical Outcomes—A Scoping Review

**DOI:** 10.3390/cancers17050863

**Published:** 2025-03-03

**Authors:** Kamil Adamczyk, Konrad Zuzda, Miłosz Jankowski, Rafał Świerczyński, Kamil Chudziński, Bartosz Czapski, Konstanty Szułdrzyński

**Affiliations:** 1 Department of Anesthesiology and Intensive Care, National Medical Institute of the Ministry of the Interior and Administration, 02-507 Warsaw, Poland; 2 Department of Neurosurgery, National Medical Institute of the Ministry of the Interior and Administration, 02-507 Warsaw, Poland

**Keywords:** cancer pain, opioids, immune response, genetic factors

## Abstract

**Background/Objectives**: Managing cancer-related pain presents complex challenges involving the interplay between analgesic efficacy, immune system responses, and patient outcomes. **Methods**: Following the Scale for the Assessment of Narrative Review Articles (SANRA) criteria, we conducted a comprehensive literature search in Medline, Scopus, and Web of Science databases. The review synthesized evidence regarding opioid pain management modalities, genetic variations affecting pain perception, and associated drug metabolism. **Results:** The literature reveals significant associations between opioid administration and immune function, with potential implications for cancer progression and survival. Genetic polymorphisms in key genes influence individual responses to pain opioid metabolism and, finally, pain management strategies. The immunosuppressive effects of opioids emerge as a critical consideration in cancer pain management, potentially influencing disease progression and treatment outcomes. **Conclusions:** Genetic variants influence analgesic efficacy, while the interaction between opioid-induced immunosuppression and genetic factors impacts both pain control and survival outcomes. This emphasizes the need for personalized treatment approaches considering individual genetic profiles and immune function.

## Simple Summary

This review examines the relationship between cancer pain management, patients’ genotype, and outcomes. The article highlights how genetic differences influence individual responses to pain treatments and opioid processing. The authors conclude that pain management in cancer care involves a delicate balance between treatment effectiveness and immune system impact, with genetic factors playing a crucial role in treatment response. To enhance the reliability of pain management practices, conducting further studies is essential for assessing the long-term effectiveness of novel genetic-based approaches.

## 1. Introduction

Oncology pain management is currently one of the key aspects of comprehensive cancer treatment, significantly contributing to better quality of life (QoL) and survival for patients. However, recent studies suggest a more complex connection between pain management and patient outcomes [1,2]. While pain relief is crucial, its potential effects on immune function and, consequently, tumor progression require careful consideration.

Studies on the interplay between pain, opioid consumption, and survival expectancy in cancer patients have profound implications for patient care. Chronic pain, a common symptom of malignancy, not only weakens patients physically but also impacts significant physiological effects that can alter the disease process and patients’ coping mechanisms [3,4]. Personalized medicine increasingly emphasizes the genetic variations that affect both the opioid efficacy and host response, thereby leading to differences in chronic pain perception, opioid effects, and therapeutic outcomes [5,6]. Genes regulating drug transport and metabolism further affect the response to treatment, adding to the challenge of tailoring pain management strategies.

This review explores the relationship between pain, opioid use, and survival in cancer patients. It highlights the significance of genotyping and the impact of opioids on immune function. The authors hypothesize that opioid-related adverse effects, particularly immunomodulation and hyperalgesia, may negatively impact survival and other clinical outcomes in cancer patients.

## 2. Methodology

This narrative review followed the Scale for the Assessment of Narrative Review Articles (SANRA) criteria [7] to comprehensively evaluate the relationships between genetic factors, pain management, and survival outcomes in cancer patients.

A literature search was conducted using Medline, Scopus, and Web of Science databases. The initial search identified over 15,000 potentially relevant studies addressing three key domains: pain perception mechanisms, opioid metabolism pathways, and cancer-related pain processes. Search terms included variations of “cancer pain genetics”, “opioid pharmacogenetics”, and “cancer pain immunology”.

The review synthesizes current evidence regarding genetic variations affecting three major pathways: pain perception, drug metabolism, and the host’s immune response. Genetic polymorphism studies were the focus concerning their effects on clinical outcomes, including the effectiveness of pain control, opioid-related adverse effects, and patient survival.

## 3. Pain Perception, Opioid Use, and Survival

**Figure 1:**
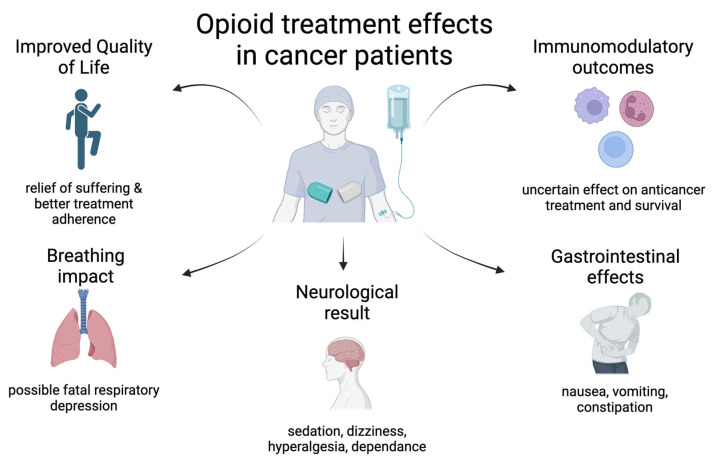
Opioid treatment effects in cancer patients—a graphical summary.

The complex interaction between pain perception, opioid usage, and overall survival outcomes is a key area of focus in oncology research, with significant implications for patient outcomes (Figure 1). Emerging evidence underscores pain perception as a critical, independent factor influencing prognosis in various oncological settings. A comprehensive systematic review by Zylla et al. revealed that pain at the time of diagnosis in advanced non-small cell lung cancer (NSCLC) was associated with shorter survival, with 80% of the analyzed studies affirming pain’s prognostic importance [1]. The data described by Zheng and colleagues indicated that pain significantly predicts survival rates in different forms of cancer, which is especially evident in prostate cancer patients [2,8]. Moreover, Reyes-Gibby’s team demonstrated that pain could serve as a key indicator of survival in head and neck cancer patients. These findings highlight the potential interplay between advanced pain management and improving cancer treatment outcomes [9]. Additionally, Boland and Bennett described the undertreatment of pain as threatening patients’ survival [10]. It is possible to improve the QoL, treatment adherence, and overall outcomes through adequate pain control, potentially extending patients’ survival [11].

The immunomodulatory effects of opioid administration represent a complex and nuanced aspect of cancer care. Recent data indicate that opioid analgesics influence cancer progression through various mechanisms. Kavgaci et al. [12] highlighted immunological interactions concerning immune checkpoint inhibitor therapies (ICIT). It was proposed that modulation of the μ-opioid receptor significantly modifies the survival mechanisms, alters cancer progression, and affects the immune system’s functioning. The activation of μ-opioid receptors in tumor and immune cells may cause tumor proliferation, suppression of apoptosis, and angiogenesis through pathways like EGFR and MAPK/ERK, among others. Opioids also inhibit the signaling of T cells by T-cell receptors, reduce CD8+ T cell effector activity, increase regulatory T cell populations, and potentially aid in forming an immunosuppressive tumor microenvironment [12]. Chancellor et al. demonstrated that prolonged opioid exposure correlates with reduced survival following lung cancer resection [13]. A comprehensive meta-analysis by Ju et al. further studied this relationship, revealing potential negative immunological consequences of opioid utilization in patients receiving ICIT [14]. Conversely, Zylberberg et al. recorded increased survival rates for elderly pancreatic cancer patients with opioid treatment [15].

The area that has evolved in recent years is the immunosuppressive effect of opioids. According to a study by Bradley and Boland [16], administration of opioids leads to immunological compromise by targeting immune cells expressing opioid receptors and through any central mechanisms that inhibit NK cell activity. These and other related mechanisms may predispose the individual to infections, including reduced cytotoxicity by NK cells and altered immune responses via opioid-induced activation of TLR-4. In addition, Shao et al. [17] reported that in advanced cancer patients, the risk of infection increased by approximately 2 percent for every 10 mg increase in the daily oral morphine equivalent (OME). It is important to note that while this association suggests a dose-dependent relationship, higher opioid dosages in these patients may also reflect a more advanced disease state or other confounding factors rather than being solely attributable to the immunomodulatory effects of opioids. Montagna et al. reported a significantly greater improvement in recurrence-free survival in patients with triple-negative breast cancer who received intraoperative administration of opioids, where fentanyl was the primary opioid given. However, hydromorphone and morphine were also included and converted to oral morphine milligram equivalents (MME) for analysis [18], while Connolly et al. conversely documented shortened progression-free survival in advanced prostate cancer with increased opioid exposure [19].

## 4. Refractory Cancer Pain: Mechanisms and Consequences

### 4.1. Mechanisms Affecting the Response to Analgesics

The management of refractory cancer pain in advanced malignancies remains a significant clinical challenge due to its multifaceted etiology and complex pathophysiology. Cancer-related pain arises through diverse mechanisms, broadly classified as nociceptive or neuropathic. Nociceptive pain typically results from tumor invasion, leading to inflammation and direct tissue damage. In contrast, neuropathic pain stems from nerve injury or dysfunction, often aggravated by tumor progression or adverse effects of treatment modalities [20]. The interaction of several biological processes primarily determines the pathophysiology of cancer pain. Tumors can secrete inflammatory mediators sensitizing the nociceptors, which results in the increased recognition of pain. For instance, the cytokines, tumor necrosis factor-alpha (TNFα), and interleukin-1 beta (IL-1β), as well as the nerve growth factor (NGF), are some of the crucial molecules in the activation and sensitization of primary afferent nociceptors. The mediators, like bradykinin and endothelin-1 (ET-1), contribute to nociception. Proteases and protease-activated receptors (PARs) modulate further the pain response by making nociceptors more excitable in the cancer microenvironment. Thus, the impact of these inflammatory mediators is very complex and lies at the basis of chronicity, which often leads to resistance to pain in the end [21,22,23,24]. Additionally, peripheral nerve injury (PNI), due to tumor invasion, can lead to neuropathic pain, which is often more challenging to treat and can significantly impair the quality of life [25].

Research indicates that central sensitization is crucial in maintaining cancer pain [26]. This phenomenon involves changes in the central nervous system, particularly within the spinal cord, where synaptic plasticity can enhance pain-signaling pathways [27]. Studies have shown that bone cancer elicits, among its mechanisms, distinctive central sensitization effects such as enhanced synaptic transmission, increased excitability of dorsal horn neurons, and plastic changes in spinal sensory pathways [28]. These alterations result in generalized changes in spinal cord excitability, leading to chronic pain and hypersensitivity. Additionally, the activation of microglia and the production of pro-inflammatory mediators such as IL-18 and phosphorylated p38 MAPK (p-p38) have also been implicated in preclinical studies to persist in cancer pains. Those mediators are upregulated in spinal microglia during advanced cancer pain and contribute to neuronal hyperactivity and central sensitization. ATP, acting through the P2X7 receptors, further increases this inflammatory cascade by microglial release of IL-18, thereby amplifying nociceptive signaling pathways [29].

### 4.2. Consequences of Pain Refractory Pain to Conventional Treatment

The clinical management of refractory cancer pain often requires a multidisciplinary approach, as standard analgesic treatments, including opioids, may prove insufficient for many patients [30,31]. Approximately 10–15% of cancer patients experience pain that remains unmanageable despite high-dose opioid therapy [32]. This necessitates the exploration of alternative therapeutic strategies, such as intrathecal drug delivery systems, which have shown promise in managing refractory pain [33].

Importantly, uncontrolled pain can lead to significant immunosuppression by activating the hypothalamic–pituitary–adrenal axis and sympathetic nervous system. This stress response increases glucocorticoid levels and catecholamines, suppressing innate and adaptive immunity [34]. In this context, effective pain management with opioids may help preserve immune function by preventing pain-induced immunosuppression, despite opioids’ direct immunosuppressive effects. While opioids can suppress immune responses directly, their ability to control pain may protect against the profound immunosuppressive effects of uncontrolled pain and chronic stress. Therefore, the net impact of opioid therapy on immune function likely depends on the balance between these competing effects in each patient.

Poorly controlled cancer pain is a significant clinical issue that can lead to a multitude of complications affecting both psychological and physical well-being, including daily functioning. Pain can interfere significantly with patients’ ability to perform routine activities, leading to increased dependency on caregivers and a diminished sense of autonomy [35,36]. Research has shown that even mild pain, rated as low as two on a zero-to-ten numerical rating scale, has been found to impair daily functioning severely [36]. Furthermore, the psychological ramifications of unmanaged pain are substantial; patients often experience heightened levels of anxiety and depression [37]. The interplay between physical pain and psychological distress creates a vicious cycle that can lead to increased healthcare utilization and costs, as patients may seek more frequent medical interventions to manage their symptoms [38].

## 5. Immunosuppression Due to Opioid Treatment

Recent studies have demonstrated that opioid therapy can contribute to immunosuppression [39], potentially worsening cancer progression and patient survival [40]; however, the extent to which these outcomes are directly attributable to the immunomodulatory effects of opioids—as opposed to other risk factors inherent to patients requiring opioid treatment—remains uncertain [41].

Opioids exert their analgesic effects by binding to seven trans-membrane G protein-coupled receptors (GPCRs)—so-called opioid receptors. These receptors are classified as classical and non-classical. The classical group includes three main subtypes: μ, κ, and δ [42]. Although opioids can exert their pharmacological effects through κ and δ opioid receptors, most opioids act mainly on μ-opioid receptors (MORs), which is their primary analgesia mechanism [43]. Opioid binding and MOR activation inhibit excitatory neurotransmitter release from presynaptic neurons while hyperpolarizing postsynaptic neurons [44]. Analgesia is primarily mediated by opioid receptors in the central nervous system (CNS), although there is evidence that opioid receptors in the peripheral nervous system may also mediate analgesia [45].

Opioid receptors are expressed in neurons, neutrophils, macrophages, and T and B lymphocytes [44]. Their widespread distribution can lead to various side effects such as sedation, constipation, nausea, pruritus, physical dependence, vomiting, dizziness, respiratory depression, hyperalgesia, and shock due to histamine release, as well as immunosuppression and increased infection risk [46,47,48]. The effects of endogenous opioids on the immune system are complex and can be immunostimulatory or inhibitory, depending on the type of cells they affect [49].

Opioids impair innate and acquired immunity by directly interacting with immune cells or indirectly activating the central nervous system and hypothalamic–pituitary–adrenal axis, releasing immunosuppressive corticosteroids and hormones [50,51,52,53,54]. Recent controlled studies substantiate these findings. For example, Chen [55] demonstrated that repeated intraperitoneal injections of oxycodone in mice significantly reduced CD4^+^ T cells dose-dependently. Similarly, McIlvriedd found that morphine treatment in tumor-bearing models diminished CD4^+^ and CD8^+^ T cell infiltration, reducing the efficacy of immune checkpoint inhibitors such as anti-PD-1; these effects were mediated via *OPRM1* signaling in CD8^+^ T cells and could be counteracted by peripherally acting *OPRM1* antagonists [56]. Supporting these preclinical findings on a broader scale, Mao et al. [57] reported that opioid use was associated with decreased response rates to immune checkpoint inhibitors (OR = 0.49) and increased risks of disease progression (HR = 1.61) and mortality (HR = 1.67).

Morphine activates MOR on various innate immune cells [50,51,58]. In macrophages, this results in reduced proliferation, recruitment, phagocytosis, and bactericidal activity, potentially via interaction with Toll-like receptors (TLRs) and altered signaling of the transcription factor NF-κB. Morphine inhibits neutrophil migratory function by interfering with IL-8 signaling and reduces neutrophil superoxide production, impairing their bactericidal activity [50,51,58]. Morphine, moreover, reduces mast cell activation, increasing intestinal permeability and the risk of infections [50,51,58].

Clinical observations lend further support to these mechanisms. Kc et al. [59] linked latent herpes simplex virus (HSV) reactivation to chronic morphine use, while Puzhko et al. [60] reported that opioid users face increased risks of infections such as hepatitis C and HIV [59,60]. Moreover, Kelty et al. [61] showed that prenatal opioid exposure may induce long-term immune dysregulation, predisposing children to diseases and conditions like eczema and asthma. Similarly, Kudrina [62] noted that chronic opioid use alters the intestinal microbiome and activates the HPA axis [61,62]. In oncology, Scarpa et al. [63] demonstrated that opioids—particularly morphine—impair the efficacy of immune checkpoint inhibitors by modulating RNA expression networks in CD8^+^ T cells, underscoring the clinical impact of opioid-induced immunosuppression on cancer therapy.

## 6. Genetic Profiling

Genetic variations can significantly impact a patient’s pain perception and response to pain medication, influencing treatment outcomes and QoL. Recent findings align with the broader applications of pharmacogenomics in pain management, supporting the development of personalized treatment protocols to optimize therapeutic efficacy while minimizing adverse effects [64]. By identifying specific genetic markers associated with pain sensitivity and analgesic response, clinicians can tailor treatments to individual patients, potentially improving pain control and reducing the risk of adverse effects [Table 1].

### 6.1. OPRM1, OPRD1, and OPRK1 Genes

The genes OPRM1, OPRD1, and OPRK1, which encode for the classical opioid receptors (μ-, δ-, and κ-), have been implicated in the modulation of pain and potentially in the survival of cancer patients. Differential expression in chronic pain or opioid addiction suggests their utility as biomarkers [65,66]. Studies examining opioid response in cancer patients have highlighted the role of genetic polymorphisms, particularly in the OPRM1 gene. Variants in this gene can affect opioid metabolism and receptor sensitivity, influencing pain management [67,68]. For example, polymorphisms such as OPRM1 rs1799971 and rs677830 can affect opioid receptor sensitivity, which may increase the risk of side effects such as constipation and alter the effectiveness of pain relief.

Although some suggest that cancer patients require high opioid doses and rapidly develop tolerance, Schug et al. [69] demonstrated effective pain control with lower doses—dose increases were more often due to disease progression. In contrast, Gagnon et al. [70] observed escalating opioid needs in advanced stages, and Preux et al. [71] reported an 8% prevalence of opioid use disorder. In this context, µ-opioid receptor downregulation [72] and genetic variations, including OPRM1 rs9479757 and OPRK1 rs7824175 [73], may contribute to differences in analgesic efficacy. Poor pain management can increase emotional distress, anxiety, and depression, all of which are known to lower QoL and potentially shorten survival in oncology patients [74,75,76].

### 6.2. NOP Gene of the Nociceptin/Orphanin FQ Receptor

Activated by nociceptin/orphanin FQ (N/OFQ), the NOP receptor is present throughout the central and peripheral nervous systems, affecting pain modulation, emotional responses, and stress regulation [77,78]. Its distinct pharmacological profile sets it apart from classical opioid receptors, making it a promising target for cancer-related pain management [79,80]. NOP receptor activation inhibits nociceptive neurotransmission by reducing cAMP production and altering calcium and potassium currents in neurons [81,82]. This is especially relevant for patients who suffer from complex pain due to tumor growth, metastasis, and treatment side effects. Selective NOP agonists, such as SR14150, SR16835, and Ro65-6570, have demonstrated significant analgesic effects in preclinical models of cancer-induced bone pain, suggesting targeted therapies that may reduce the side effects of traditional opioids [83,84]. NOP receptors located on spinal microglia cells play a key role in neuroinflammatory processes associated with cancer pain by regulating microglia activation and the production of pro-inflammatory cytokines. This modulation affects pain-signaling pathways, thereby contributing to the development and maintenance of cancer-related pain [85]. Modulating microglial activity through NOP signaling could mitigate pain and underlying inflammation in chronic cancer pain states [86]. In addition, combining NOP and µ-opioid receptor pathways using bifunctional ligands such as cebranopadol, buprenorphine, AT-121, or SR 16435 can increase analgesic efficacy through synergistic effects on both receptors. At the same time, this combination can reduce typical opioid side effects, such as tolerance development and addiction, making pain therapies safer and more effective [78,83].

Polymorphisms in the NOP gene can alter receptor expression or function, influencing pain experiences and analgesic efficacy [87]. Beyond pain modulation, NOP receptors regulate emotional and stress responses, which are often intensified in cancer patients [77,80]. NOP modulators also exhibit anxiolytic and antidepressant properties, benefiting cancer patients with anxiety and depression [79,87].

### 6.3. CACNA1B Gene

CACNA1B gene encodes the N-type calcium channel subunit CaV2.2. This gene is integral to the functioning of voltage-gated calcium channels, essential for neurotransmitter release in the nervous system, particularly in pain pathways [88]. The modulation of calcium influx through these channels is crucial for synaptic transmission and neuronal excitability, which are fundamental in processing pain signals [88]. The expression levels and functional variants of CACNA1B may influence how patients perceive pain and respond to analgesic treatments. For instance, overexpression of CACNA1B has been associated with enhanced neuronal excitability, which can exacerbate pain sensations and hyperalgesia [11]. In addition, specific genetic variants in the CACNA1B gene may alter opioid efficacy by affecting alternative splicing of CaV2.2 calcium channels. These variants may modify DNA methylation, which affects CTCF protein binding and, ultimately, the expression of more opioid-sensitive channel isoforms [89]. Activating MORs can inhibit N-type calcium channel activity, thereby reducing calcium influx and neurotransmitter release, a critical mechanism for pain modulation. Variations in the expression or function of CACNA1B may disrupt this balance, leading to altered responses to opioid analgesics and necessitating adjustments in pain management strategies for cancer patients [90].

### 6.4. BCL2 and BAX (Apoptosis-Related Genes)

BCL2, an anti-apoptotic gene, and BAX, a pro-apoptotic gene, maintain a balance that affects cellular responses to stress and pain. Their expression levels modulate pain pathways, where increased BAX and decreased BCL2 are associated with heightened pain sensitivity and chronic pain development. Pro-inflammatory cytokines like IL-1β and TNF-α upregulate BAX and downregulate BCL2, enhancing neuronal apoptosis and pain perception [91,92,93]. The activation of microglia and the subsequent release of inflammatory mediators exacerbate this process, creating a vicious cycle of pain and inflammation [93]. In glial cells, dysregulation of BCL2 and BAX contributes to neuroinflammation and pain hypersensitivity in neuropathic pain models [94]. Targeting the BCL2/BAX pathway has been shown to reduce glial activation and pain behaviors [95,96]. Cancer treatments, particularly chemotherapy, often induce neuropathic pain, significantly impairing patients’ QoL. Thymoquinone and mangiferin alleviate neuropathic pain by modulating the BCL2/BAX pathway, promoting neuronal survival, and reducing apoptosis [95,97]. Genetic polymorphisms in BCL2 and BAX influence individual pain sensitivity and responses to analgesic treatments, highlighting the potential for personalized pain management strategies [98]. In addition, non-pharmacological approaches such as electroacupuncture affect BCL2 and BAX expression by regulating the miR-206-3p/BDNF pathway, reducing levels of the pro-inflammatory cytokines IL-6 and TNF-α, and modulating apoptosis pathways, which offers integrative approaches to cancer pain management [99,100].

### 6.5. FAAH (Fatty Acid Amide Hydrolase) Gene

Fatty Acid Amide Hydrolase (FAAH) is essential for metabolizing endocannabinoids like anandamide, which modulates pain and inflammation. This makes FAAH inhibition a promising strategy for cancer pain management [101,102]. FAAH is responsible for the degradation of anandamide, an endocannabinoid that interacts with cannabinoid receptors to exert analgesic effects. Elevated levels of anandamide have been associated with reduced pain sensitivity, suggesting that FAAH inhibition may enhance analgesia by increasing the availability of this endocannabinoid. Cajanus et al. [103] demonstrated that common single-nucleotide polymorphisms (SNPs) in the FAAH gene can influence pain sensitivity in postoperative settings, indicating a genetic basis for individual variability in pain response. Moreover, the FAAH’s role in pain modulation is further illustrated by the discovery of FAAH-OUT, a novel pseudogene that may regulate *FAAH* expression. FAAH-OUT expression could significantly change anandamide levels, affecting pain sensitivity and response to analgesics [104]. In a study by Nasirinezhad and team, FAAH inhibition significantly attenuated persistent pain-related behaviors in a rat model of human immunodeficiency virus (HIV) sensory neuropathy, suggesting that FAAH inhibitors could be beneficial for managing pain in diverse clinical contexts [105].

### 6.6. KCNJ6 (GIRK2) Gene

The KCNJ6 gene encodes the G-protein-regulated inward-rectifier potassium channel-2 (GIRK2) potassium channel. Polymorphisms in the KCNJ6 gene affect pain responses by modifying the function of GIRK2 channels that regulate opioid receptors, leading to changes in pain tolerance and opioid efficacy and affecting treatment outcomes and quality of life for cancer patients [102]. Variants in KCNJ6 are associated with pain-related phenotypes, such as pain intensity and opioid analgesic effectiveness. Ozberk et al. found that the KCNJ6 polymorphism rs2070995 affects methadone response in advanced cancer patients [106]. Elens et al. further demonstrated that specific KCNJ6 polymorphisms, along with other genetic factors, can lead to inadequate opioid-induced analgesia in preterm infants [107]. Langford’s study showed that variations in potassium channel genes, including KCNJ6, are associated with preoperative breast pain in women with breast cancer [108]. Smith’s review of potassium channels in neuropathic pain underscored KCNJ6’s broader implications in pain management, suggesting that targeting sensory abnormalities linked to KCNJ6 could improve analgesic efficacy [109]. Jeremic et al. discussed the therapeutic potential of GIRK channels in the central nervous system, proposing that modulating these channels can enhance pain pathway regulation and analgesic outcomes for cancer patients [110]. Additionally, Bony et al. [111] highlighted the interaction between GIRK channels and GABA_B receptors, indicating that activating GIRK channels via GABA_B agonists can reduce neuronal excitability and manage pain effectively.

### 6.7. CYP3A4 (Cytochrome P450 3A4) Gene

Cytochrome P450 3A4 (CYP3A4) is crucial for metabolizing various opioid analgesics, including fentanyl (to norfentanyl), sufentanil, alfentanil, and methadone through N-demethylation pathways. Genetic polymorphisms and environmental factors lead to significant variability in CYP3A4 activity, resulting in heterogeneous opioid plasma concentrations and diverse therapeutic outcomes [112]. In fentanyl metabolism, CYP3A4 facilitates the conversion to norfentanyl, significantly affecting fentanyl clearance and efficacy [113]. While CYP2B6 primarily metabolizes methadone, CYP3A4 contributes substantially to its biotransformation, influencing its pharmacokinetic profile and therapeutic effects [114]. Pharmacological interactions significantly impact CYP3A4-mediated opioid metabolism. CYP3A4 inhibitors such as ketoconazole can substantially elevate fentanyl plasma concentrations, potentially precipitating respiratory depression [113]. The interindividual variability in CYP3A4 activity, influenced by genetic polymorphisms and environmental factors, including dietary components, significantly impacts therapeutic responses [115].

### 6.8. CYP3A5 (Cytochrome P450 3A5) Gene

Cytochrome P450 3A5 (CYP3A5) is integral to the metabolism of several opioid analgesics, including alfentanil, fentanyl (via N-dealkylation), and methadone (via N-demethylation), as well as other therapeutic agents like midazolam and immunosuppressants [115]. Official international guidelines already include recommendations for adjusting the dosage of the immunosuppressant drug tacrolimus based on the CYP3A5 genotype [116]. Genetic variations in this gene result in distinct metabolizer phenotypes, leading to significant differences in the pharmacokinetics of opioids such as methadone and buprenorphine. Individuals with specific CYP3A5 variants may require tailored therapeutic adjustments to achieve optimal analgesic efficacy [112,117]. Notably, the CYP3A5*1 and CYP3A5*3 alleles are associated with functional enzyme expression, significantly impacting drug metabolism and therapeutic outcomes [118]. Patients with a single nucleotide polymorphism (SNP) of the CYP3A5*3 gene had plasma fentanyl concentrations approximately twice as high as patients with the wild-type gene polymorphism (*1*1) or patients with heterozygous gene polymorphism (*1*3) [119]. Personalized analgesic protocols based on CYP3A5 genotypes can enhance therapeutic precision and efficacy, particularly in populations with significant genetic variability affecting enzymatic activity [116,118].

### 6.9. CYP2C19 (Cytochrome P450 2C19) Gene

Cytochrome P450 2C19 (CYP2C19) is pivotal in metabolizing various pharmaceuticals, including proton pump inhibitors like omeprazole, antidepressants such as citalopram and imipramine, and anticonvulsants like S-mephenytoin. Additionally, CYP2C19 metabolizes barbiturates, diazepam, propranolol, clopidogrel, and other therapeutic agents [120,121]. In opioid metabolism, CYP2C19 facilitates the N-demethylation of methadone to EDDP, with genetic variants impacting metabolic efficiency and safety [122]. Genetic polymorphisms within the CYP2C19 gene manifest in distinct metabolic phenotypes, precipitating substantial pharmacodynamic responses and safety profile variations. Specific genetic variants, including CYP2C19*2* and *CYP2C19*3, result in reduced enzymatic activity, categorizing individuals as poor metabolizers [123].

### 6.10. UGT2B7 (UDP Glucuronosyltransferase Family 2 Member B7) Gene

The *UGT2B7* gene influences pain perception and therapeutic responses. Polymorphisms like rs7439366 are associated with variations in opioid efficacy and sensitivity, notably for morphine and fentanyl [124,125]. For instance, carriers of the mutant allele of rs7439366 exhibit a higher affinity for morphine metabolites, specifically morphine-3-glucuronide (M3G) and morphine-6-glucuronide (M6G), which make them more sensitive to morphine [126]. Sastre et al. [127] found that UGT2B7*2 homozygotes exhibit higher morphine-6-glucuronide (M6G) to morphine ratios, enhancing analgesic potency [127]. Similarly, Muraoka et al. demonstrated that UGT2B7 variants affect fentanyl dosage requirements during painful procedures [124]. Ning et al. linked the UGT2B7 C802T polymorphism to morphine efficacy, highlighting genotyping’s potential to optimize opioid therapy [128]. Margarit et al. observed genotype-dependent effects of UGT2B7 on opioid titration and pain intensity, though Gregori et al. noted COMT polymorphisms as stronger predictors of postoperative morphine needs, emphasizing the multifactorial nature of pain management [129,130]. UGT2B7 also impacts oxycodone metabolism. Li et al. reported superior pain relief in oxycontin users with the UGT2B7 802CC genotype compared to 802TT carriers, reinforcing the gene’s role in opioid pharmacogenomics [131].

### 6.11. COMT (Catechol-O-Methyltransferase) Gene

Catechol-O-methyltransferase (COMT) is pivotal in pain management by metabolizing catecholamines—dopamine, norepinephrine, and epinephrine that influence pain perception and analgesic response. Genetic variations in COMT, particularly the single nucleotide polymorphism (SNP) rs4680, are associated with differences in pain sensitivity and opioid requirements among individuals [132,133,134]. Patients with the COMT rs4680 GG genotype, associated with the higher enzymatic activity of COMT responsible for metabolizing catecholamines such as dopamine, norepinephrine, and epinephrine, generally require higher doses of opioids for effective pain control compared to those with the AA genotype [132,133]. COMT polymorphisms influence not only opioid efficacy but also overall pain experience. Patients with the low-activity AA genotype may exhibit increased pain sensitivity, complicating pain management [135,136]. Conversely, those with the high-activity GG genotype might experience reduced pain responses, risking under-treatment if their higher opioid needs are not addressed [132]. Khan’s research highlighted the significance of the COMT rs4680 SNP in predicting responses to tramadol, a commonly used postoperative analgesic, indicating that genetic testing can optimize analgesic selection and dosing [134,137]. Similarly, the RELIEF study demonstrated that genotyping for COMT variants can predict the most effective opioid for individual patients, improving pain control and minimizing side effects [132,138]. Anxiety and fear of pain have been shown to moderate the relationship between COMT genotypes and pain outcomes, particularly in chronic pain conditions like fibromyalgia [139].

### 6.12. ABCB1 (ATP-Binding Cassette Sub-Family B Member-1) Gene

The ABCB1 gene, or multidrug resistance protein 1 (MDR1), encodes P-glycoprotein, an essential ATP-dependent efflux transporter critical for opioid pharmacokinetics [140,141,142]. These differential therapeutic responses correlate with P-glycoprotein functional variations, which modulate opioid penetration across the blood-brain barrier, thereby influencing central analgesic effects [143]. Specific genetic variants in the ABCB1 gene associated with increased susceptibility to opioid-induced respiratory depression include the CT and TT genotypes of the 062rs1045642 (C > T) polymorphism. These variants correlate with adverse reactions to oxycodone, with the T allele associated with a higher incidence of such effects [144,145]. This genotype-phenotype correlation emphasizes the fundamental importance of pharmacogenetic assessment in optimizing individual analgesic protocols while minimizing adverse effect profiles [146,147]. The ABCB1 C3435T polymorphism in perioperative medicine significantly affects postoperative pain and analgesic requirements. TT genotype carriers experience lower pain intensity and require fewer opioids compared to CC genotype individuals, supporting the use of genetic profiling for personalized pain management [148]. Beyond opioids, ABCB1 polymorphisms influence responses to non-steroidal anti-inflammatory drugs and other analgesics, impacting chronic pain management and adjunctive therapies [143,149]. Moreover, ABCB1 genetic variations affect nociceptive processing and opioid dependence pathways, aiding in the development of strategies to reduce dependence risk while optimizing pain relief [5,143].

### 6.13. SLC6A3 and SLC6A4 (Solute Carrier Family 6 Member 3 and 4) Gene

The SLC6A3 and SLC6A4 genes encode the dopamine and serotonin transporters, respectively, playing crucial roles in nociceptive processing and opioid therapeutic efficacy. SLC6A3 facilitates presynaptic dopamine reuptake and is integral to nociceptive signaling cascades. A study by Qadri et al. [150] demonstrated that specific polymorphic variants in SLC6A3 significantly influence acute nociceptive intensity following traumatic events, such as vehicular collisions, highlighting the direct correlation between dopaminergic transport and nociceptive processing. Similarly, SLC6A4, which encodes the serotonin transporter protein (5-HTT), plays a significant role in modulating both nociceptive processing and the associated emotional responses. By regulating serotonin reuptake from the synaptic cleft, 5-HTT influences the intensity and duration of serotonergic signaling, thereby affecting the transmission of pain signals within the central nervous system. Genetic variations in SLC6A4 can also alter serotonin availability, impacting mood regulation and the emotional perception of pain [151].

### 6.14. DRD2 (Dopamine Receptor D2) Gene

Dopamine receptor D2 (DRD2) is essential for nociceptive processing and opioid responses. The 957C > T variant significantly influences thermal pain sensitivity, with 957TT homozygotes exhibiting lower thermal detection thresholds, indicating heightened sensitivity [152]. The DRD2 SNP rs6276 also correlates with acute pain intensity, as A/A genotype carriers report lower pain scores than A/G or G/G individuals [150]. In opioid therapy, DRD2 polymorphisms show mixed associations with outcomes. Studies in opioid-dependent patients undergoing methadone maintenance therapy found no significant links between DRD2 variants and pain, withdrawal, or sleep parameters, suggesting potential population-specific effects [153]. Patients with DRD2 variations [150] may benefit from personalized analgesic protocols, including dopaminergic modulators within opioid-sparing regimens [154].

### 6.15. NLRs (NOD-like Receptors) Gene

Nucleotide-binding oligomerization domain-like receptors (NOD-like) receptors (NLRs), particularly the NLRP3 inflammasome, are key mediators in immune and pain pathways. Activation of the NLRP3 inflammasome in spinal microglial cells triggers the release of pro-inflammatory cytokines like IL-1β, amplifying nociceptive signaling and contributing to neuropathic pain [155]. Cellular stress and injury activate NLRP3, promoting secretion of IL-1β and IL-18, which heighten pain perception and are associated with hyperalgesia, including opioid-induced hyperalgesia [156]. NLRs pathways are integral to the development of opioid tolerance, where prolonged opioid use paradoxically increases pain sensitivity. Prolonged opioid exposure activates NLRs, particularly the NLRP3 inflammasome, leading to the production of pro-inflammatory cytokines that increase pain perception. This inflammatory response mediated by NLR activation may counteract the analgesic effects of opioids. Zare’s work highlights these NLR pathways as potential therapeutic targets to reduce hyperalgesia and improve opioid efficacy, suggesting that inhibiting NLR activation may improve pain outcomes [156]. Basu et al. identified microRNAs that downregulate NLRP3 expression, suggesting therapeutic potential in neuropathic pain management [157].

### 6.16. PTGS2 (Prostaglandin-Endoperoxide Synthase 2) Gene

The PTGS2 gene, encoding cyclooxygenase-2 (COX-2), plays a central role in pain perception and inflammation by regulating prostaglandin synthesis [158]. PTGS2 expression increases significantly after surgical interventions, with higher levels correlating with greater reported pain intensity. Genetic polymorphisms in PTGS2, such as rs1799964 and rs5277, influence pain sensitivity and therapeutic outcomes. These variants are linked to enhanced pain sensitivity, particularly in lung cancer survivors, and affect the efficacy of NSAID-based analgesics by modulating PTGS2 expression patterns [159,160]. Therapeutic inhibition of PTGS2 has demonstrated efficacy across various pain conditions. Meloxicam is an example that shows antiallodynic effects in diabetic neuropathy models [161]. PTGS2 is also implicated in complex regional pain syndrome (CRPS), where its expression correlates with inflammation-driven pain amplification [162]. Regulation of PTGS2 involves interactions with transcription factors such as ATF3, a negative regulator during acute inflammation, highlighting potential targets for therapeutic intervention [163].

## 7. Clinical Applications and Treatment Implications

The genetic variations discussed in this review have significant implications for clinical practice in cancer pain management. Understanding a patient’s genetic profile can help predict both analgesic response and the risk of adverse effects, enabling more personalized treatment approaches. For example, patients with the OPRM1 rs1799971 A/A genotype [67,68] typically require lower opioid doses compared to G allele carriers, while those with CYP2D6 poor metabolizer status may need alternative analgesics instead of codeine or tramadol due to reduced drug activation. Genetic testing for COMT rs4680 [134,137] can guide initial opioid dosing, as GG genotype carriers generally require higher doses for effective pain control than AA carriers.

Implementation of genetic screening in clinical practice has shown promising results. Several studies report that genotype-guided therapy leads to better pain control and fewer adverse effects. For instance, the RELIEF study [132,138] demonstrated that selecting between morphine and oxycodone based on the COMT genotype improved pain outcomes. Similarly, screening for ABCB1 polymorphisms [5,143] has helped identify patients at higher risk for opioid-induced respiratory depression, allowing for more careful monitoring and dose adjustment.

However, genetic testing should be considered within the broader context of patient care. Age, organ function, comorbidities, and concurrent medications remain crucial in treatment decisions. The cost-effectiveness and availability of genetic testing must also be considered, though advancing technology continues to make testing more accessible. Integration of genetic information into electronic health records and clinical decision support systems could facilitate the practical application of pharmacogenetic data in routine cancer pain management.

## 8. Conclusions

The evidence demonstrates that genetic variants in opioid receptors, drug metabolism enzymes, and pain perception modulators significantly influence analgesic efficacy in cancer patients. The complex interaction between opioid-induced immunosuppression and genetic factors affects both pain control and survival outcomes, highlighting the importance of personalized treatment approaches that consider individual genetic profiles and immune system function.

## Figures and Tables

**Table 1:** Influence of Genes on Pain Perception, Opioid Therapy, and Analgesic Drugs.

Category	Gene/ Citation	Role	Impact on Pain Perception	Effect on Analgesic Drugs
Opioid Signaling Pathway	*OPRM1 OPRD1 OPRK1*	Encode classical opioid receptors μ-, δ-, and κ-, mediating opioid analgesic effects.	Variations may lead to differences in analgesic efficacy and pain control.	Influence opioid responsiveness, potentially causing inadequate pain relief or increased sensitivity.
	*NOP*	Encodes nociceptin/orphanin FQ receptor, involved in non-classical opioid signaling.	Modulates nociceptive neurotransmission, reducing pain perception.	Potential target for novel analgesics with fewer side effects compared to traditional opioids.
Pain Perception Modulators	*CACNA1B*	Encodes N-type calcium channel subunit, critical for neurotransmitter release.	Overexpression enhances pain sensation; linked to hyperalgesia.	Genetic variants may alter opioid efficacy; dysfunction affects analgesic responses.
	*BCL2 BAX*	Regulate cellular apoptosis; *BCL2* gene is anti-apoptotic, while BAX is pro-apoptotic.	Imbalance increases neuronal sensitivity to pain.	Targeting these pathways may reduce pain and modulate glial activation.
	*FAAH*	Enzyme degrading endocannabinoids, particularly anandamide.	Inhibition increases anandamide levels, reducing pain sensitivity.	*FAAH* ^1^ inhibitors may enhance analgesic effects; polymorphisms affect treatment responses.
	*KCNJ6*	Encodes GIRK2 ^2^ potassium channels, regulating neuronal excitability.	Variants influence pain sensitivity and opioid requirements.	Genetic variants may necessitate opioid dosage adjustments for effective analgesia.
Drug Metabolism Genes	*CYP2D6 CYP2B6 CYP3A4 CYP3A5 CYP2C19*	Enzymes involved in Phase I drug metabolism, including opioids.	No direct influence on pain perception.	Variants affect opioid metabolism, altering efficacy and risk of side effects like toxicity or inadequate relief.
	*UGT2B7*	Enzyme responsible for Phase II glucuronidation of opioids like morphine.	No direct influence on pain perception.	Polymorphisms impact opioid efficacy by altering metabolite levels; dosage adjustments may be required.
	*COMT*	Metabolizes catecholamines, affecting neurotransmitter levels.	Polymorphisms influence pain sensitivity and stress response.	Variants affect opioid dosage requirements; specific genotypes need higher or lower doses for effective pain control.
Transport and Regulatory Genes	*ABCB1*	Encodes P-glycoprotein, an efflux transporter affecting drug distribution and blood-brain barrier crossing.	Variants influence nociceptive processing and pain sensitivity.	Alters opioid transport, affecting drug efficacy and toxicity.
	*SLC6A3 SLC6A4*	Encode dopamine and serotonin transporters, regulating synaptic neurotransmitter levels.	Polymorphisms may influence pain modulation and emotional responses.	May affect efficacy of drugs modulating serotonergic or dopaminergic systems.
	*DRD2*	Encodes dopamine D2 receptor, involved in dopaminergic signaling.	Variants affect pain sensitivity and stress responses.	May influence opioid efficacy and risk of addiction.
	*NLRs*	Encode NOD-like receptors ^3^, involved in immune and inflammatory responses.	Activation amplifies nociceptive signaling through pro-inflammatory cytokines.	Potential therapeutic targets for reducing opioid tolerance and opioid-induced hyperalgesia.
Inflammatory Response Gene	*PTGS2*	Encodes COX-2 ^4^, essential for prostaglandin synthesis in inflammatory responses.	Variants linked to heightened pain sensitivity and inflammation.	Target for NSAIDs ^5^; polymorphisms may affect anti-inflammatory drug efficacy.

^1^ FAAH—Fatty-acid amide hydrolase-1, ^2^ GIRK2—G-protein-regulated inward-rectifier potassium channel-2, ^3^ NOD-like receptors—nucleotide-binding oligomerization domain-like receptors, ^4^ COX-2—Cyclooxygenase-2, ^5^ NSAIDs—Nonsteroidal Anti-Inflammatory Drugs.
